# Does Aerobic Vaginitis Have Adverse Pregnancy Outcomes? Prospective Observational Study

**DOI:** 10.1155/2020/5842150

**Published:** 2020-01-18

**Authors:** Mahmoud F. Hassan, Nancy M. A. Rund, Osama El-Tohamy, Mahmoud Moussa, Yahia Z. Ali, Nehal Moussa, Ahmed A. Abdelrazik, Enas A. A. Abdallah

**Affiliations:** ^1^Department of Obstetrics and Gynecology, Faculty of Medicine, Ain Shams University, Cairo, Egypt; ^2^Department of Obstetrics and Gynecology, Bugshan Hospital, Jeddah, Saudi Arabia; ^3^Department of Obstetrics and Gynecology, Faculty of Medicine, Fayoum University, Fayoum, Egypt; ^4^Laboratory Department, Ministry of Interior Hospitals, Cairo, Egypt; ^5^Department of Obstetrics and Gynecology, Nasser Institute for Research and Treatment, Cairo, Egypt; ^6^Department of Pediatrics and Neonatology, Faculty of Medicine, Cairo University, Cairo, Egypt

## Abstract

**Background:**

Aerobic vaginitis (AV) is an aberration within the balanced vaginal microbiota. Only few reports have documented the adverse pregnancy outcomes related to AV. Nonetheless, the exact role of AV in pregnancy and the potential benefit of its screening need further study. Our goal was to evaluate the association between aerobic vaginitis (AV) in late pregnancy and maternal and neonatal outcomes.

**Methods:**

In this prospective observational study, a total of 600 singleton pregnant women with intact fetal membranes at a gestational age of 34-36 weeks were recruited (one hundred women with AV and 500 pregnant women without AV). The study protocol excluded patients with other forms of vaginal infection. Pregnancy outcomes were traced and documented. The primary outcome was the association between AV and preterm labor. The current study compared the maternal and neonatal outcomes among pregnant women with and without AV in unadjusted and adjusted analyses with the odds ratio (OR) and 95% confidence interval (CI) reported.

**Results:**

There was an association between AV and with preterm birth (adjusted OR 3.06, 95% CI 1.58-5.95) and prelabor rupture of membranes (adjusted OR 6.17, 95% CI 3.24-11.7). For neonatal outcomes, AV was associated with a higher incidence of neonatal ICU admission (adjusted OR 2.19, 95% CI 1.1-4.34). Severe forms of AV significantly increased the incidence of PTB (*p* = 0.0014) and PROM (*p* = 0.0094) when compared to less severe forms of AV.

**Conclusion:**

AV is common in late pregnancy and is linked to a diversity of adversative pregnancy outcomes including preterm birth, PROM, and neonatal ICU admission. Moreover, the incidence of PTB and PROM might further increase with the severity of AV. Clinicians should pay more consideration to vaginal microbiota assessment during pregnancy.

## 1. Introduction

The vaginal bacterial flora consists of many microbial species, principally Lactobacillus species. The balance and interactions among vaginal microbes are critical for a healthy vaginal microenvironment [1]. Physiological changes during pregnancy result in vaginal mucosal congestion and hypertrophy, which benefit the growth of pathogenic microorganisms within the vagina [2]. The definition of aerobic vaginitis (AV) is a clinical entity first proposed by Donders et al. in 2002 [3]. Aerobic vaginitis is an aberration within the balanced vaginal bacterial flora. Aerobic vaginitis is characterized by abnormal vaginal microflora accompanied by an increased localized inflammatory reaction and immune response [4]. The prevalence of AV varies from 5 to 10.5% among symptomatic nonpregnant women [5] and 4 to 8% during pregnancy [6]. Concerns about AV have been raised because it tends to involve mixed infections [7]. If undiagnosed or untreated, AV might interfere with female reproductive health and result in perinatal complications, such as preterm birth (PTB), prelabor rupture of membranes (PROM), and fetal infection [8, 9].

Notwithstanding the association between AV and pregnancy outcomes has been investigated in several studies, AV remains incompletely understood [10]. Previous epidemiological studies recognized a high incidence of an unexpected pregnancy outcome and premature birth due to AV in Africa and worldwide [10, 11]. In attempts to explore the association between AV in late pregnancy and adverse perinatal outcomes, we conducted this prospective, observational, and powered study.

## 2. Materials and Methods

We conducted the present prospective observational study at the Obstetrics and Gynecology Department, Bugshan Hospital, Jeddah, Saudi Arabia, from February 2013 till November 2018. The Local Institutional Review Board and Ethics Committee granted the study protocol before study commencement. All participants provided written consent before inclusion. Our study followed the ethical standards of the Declaration of Helsinki. All pregnant women at a period of gestation 34-36 weeks as calculated by the last menstrual period or the first trimester ultrasound diagnosed with AV were consecutively enrolled. Participants were selected among pregnant women who attended for routine antenatal care in the outpatient clinic. We excluded women with history of previous preterm labor or threatened preterm labor, multiple gestation, women with rupture of membranes, antepartum hemorrhage, structural and functional abnormalities of the uterus, other specific vaginal infection including vulvovaginal candidiasis (VVC), bacterial vaginosis (BV), Trichomonas vaginalis (TV), or mixed vaginal infections (≥2 types of simultaneous vaginal infection), and induced preterm labor for any obstetrical and medical condition.

At enrollment, all participants had detailed history, clinical examination, and a detailed transabdominal sonography. Then, participants underwent gynecological examinations and vaginal discharge collection. A nonlubricated sterile speculum was inserted before any other vaginal examination was made. Vaginal pH was evaluated by color strips. We obtained samples of vaginal discharge from the upper lateral vaginal wall using long cotton swabs. Samples were spread in the clinic onto three slides and were mixed with a drop of saline on one slide and a drop of 10% potassium hydroxide (KOH) on a second slide; the third slide was Gram-stained. Then, all slides were sent for immediate microscopic examination. Also, one vaginal swab sample was sent to the laboratory for further aerobic culturing aimed at detection of aerobic bacterial growth.

Aerobic vaginitis was diagnosed when a composite AV score ≥ 3 was determined by saline wet mount microscopy [3]. We used Nugent's criteria to diagnose BV based on Gram stain assessment [12]. Candidiasis was determined by direct observation of hyphae or budding yeast on a 10% potassium hydroxide preparation slide [13]. Trichomonas vaginalis was diagnosed when TV was microscopically distinguished in the saline wet mount smear [14]. All smears were examined by the same microscopist who was blinded for the patient's data. Confirmed cases with lone AV and control participants were followed up to evaluate pregnancy outcomes.

The primary outcome was preterm birth (defined as delivery before 37 weeks). Secondary outcome measures encompassed the following maternal outcomes: prelabor rupture of membranes (PROM) (defined as rupture of membranes before the onset of labor), preterm PROM (defined as rupture of membranes before 37 weeks), chorioamnionitis, cesarean delivery, and puerperal sepsis. The following neonatal outcomes were considered: low birth weight (defined as birth weight < 10th percentile for gestational age) [15], neonatal jaundice, neonatal sepsis (confirmed with a positive culturing for microorganisms from a sample of CSF, blood, or urine) [16], neonatal asphyxia (defined as (1) arterial cord pH < 7.0, (2) Apgar score of 3 or less for greater than 5 minutes, (3) evidence of altered neurological status, and (4) multisystem organ injury or failure) [17], neonatal intensive care unit (NICU) admission, stillbirth (defined as a baby born with no signs of life at or after 28 weeks of gestation) [18], and neonatal death (defined as deaths among live births during the first 28 completed days of life) [18].

The perinatal outcomes were compared between the offspring of patients with aerobic vaginitis and without AV. Baseline clinical characteristics between women with AV and without AV were compared using the chi-squared test or Fisher exact test for categorical variables and the Mann-Whitney *U* test or Student *t*-test for continuous variables, as appropriate. Odds ratios (ORs) and 95% confidence intervals (CIs) were calculated for the outcomes of interests. Multivariate logistic regression was used to adjust for patients' age, BMI, parity, previous cesarean delivery, hypertensive disorders with pregnancy, pregestational and gestational diabetes mellitus, smoking habits, education level, and woman's occupation. Normality of distribution of the continuous variables was evaluated using the Shapiro–Wilk test. We tested final models with the Hosmer-Lemeshow goodness-of-fit test. All analyses were performed using the Statistical Package for the Social Sciences, version 14.0 (SPSS Inc., Chicago, IL, USA), and GraphPad Prism, version 6 (GraphPad Software Inc., La Jolla, CA, USA).

A priori sample size estimation was performed using PS: Power and Sample Size Calculation, version 3.1.6 (Department of Biostatistics, Vanderbilt University, Nashville, TN, USA). We planned a study of independent cases and controls with five controls per one case to study the association between AV and preterm birth (primary outcome). Prior data show that the PTB rate among controls was 9.2% and 19.7% for experimental subjects [19]. Thus, we need to study at least 95 experimental subjects and 475 control subjects to reject the null hypothesis that the PTB rates for experimental and control subjects are equal with a power of 80% and a type I error of 0.05.

## 3. Results

**Figure 1:**
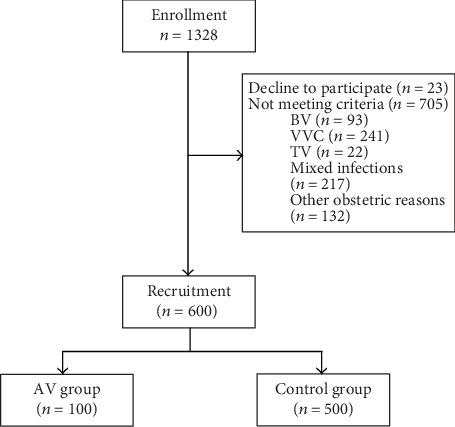
Patient flow chart. BV: bacterial vaginosis; VVC: vulvovaginal candidiasis; TV: Trichomonas vaginalis; AV: aerobic vaginitis.

Figure 1 represents the patient flow chart. Initial recruitment included 1,328 women. Twenty-three women declined to participate in the study. Meanwhile, 705 women did not match the criteria for participation in our study.

Several clinical characteristics were similar between pregnant women with aerobic vaginitis and pregnant women without AV in our cohort including age, BMI, parity, previous cesarean delivery rate, hypertensive disorders with pregnancy, pregestational and gestational diabetes mellitus, and smoking habits. However, a few characteristics have differed between both groups. Women with AV were less likely to have college-level education or above and were more likely to be not working (Table 1).

In unadjusted analysis, there was an association between women with AV and preterm birth (18% vs. 6.8%) and prelabor rupture of membranes (24% vs. 5.4%) and neonatal asphyxia (6% vs. 1.6%) compared with women without AV (Table 2). In multivariate analysis, pregnant women with AV remained associated with preterm birth (adjusted OR 3.06, 95% CI 1.58-5.95) and prelabor rupture of membranes (adjusted OR 6.17, 95% CI 3.24-11.7) (Table 2).

For neonatal outcomes, pregnant women with AV were associated with neonatal asphyxia (5% vs. 1.6%) and neonatal ICU admission (15% vs. 7.2%) compared with pregnant women without AV in unadjusted analysis (Table 3). In the adjusted analysis, women with AV remained associated with neonatal ICU admission (adjusted OR 2.19, 95% CI 1.1-4.34) compared with pregnant women without AV. However, women with AV were no longer significantly associated with neonatal asphyxia in the adjusted analysis (Table 3).

Aerobic culturing revealed that the most commonly identified pathogens among symptomatic women with a presumptive diagnosis of AV were Staphylococcus aureus, Enterococcus faecalis, Streptococcus agalactiae, and Escherichia coli. Other less frequent isolated pathogens were Staphylococcus saprophyticus, Lactobacillus acidophilus, Staphylococcus epidermidis, Corynebacterium, and Streptococcus pyogenes.

Table 4 provides information about the comparison of the maternal and neonatal outcomes according to the severity of AV. It indicates that the incidence of PTB and PROM is significantly increased with the severity of AV.

## 4. Discussion

The equilibrium and interactions between vaginal microbes are important to female vaginal health. Lactobacilli that dominate within the vaginas of most healthy women play a significant role in protecting the host from genital tract infections [20, 21]. Aerobic vaginitis is a newly recognized vaginal flora disorder characterized by a shift from vaginal microbiota dominated by the Lactobacilli to an overgrowth of aerobic bacteria leading to adverse perinatal outcomes [22].

In our study, screening pregnant women for AV at 34-36 weeks of gestation declared higher odds for preterm labor, PROM, and neonatal ICU admission in pregnant women with AV than control participants. Moreover, severe forms of AV significantly increased the incidence of PTB and PROM. Ascending infection caused by vaginal microorganisms might justify the mechanism of AV-related adverse pregnancy outcomes. Some bacterial species in AV produce sialidases, which degrade host defense molecules such as IgA and can remove sialic acid from mucosal epithelial cells and mucins. The elimination of sialic acid from secretory IgA leads to IgA proteolysis and a lowered local immune response [23]. On top of that, AV probably linked to the increased concentrations of IL-1b, IL-6, and IL-8 which are known risk factors for adverse pregnancy outcome [6, 24].

Many earlier studies have not recognized or ignored the contributing effect of AV on perinatal outcomes [25, 26]. However, new culture-based studies confirmed the association between AV and preterm birth [4, 27]. On the contrary, a recent study by Han and coworkers did not show the association between AV and preterm labor. They incorporated cases with mixed vaginal infection along with many cases with solitary vaginal infection pathogens other than AV in their study; thus, they had a smaller population with only AV. Nevertheless, the same study was compatible with our findings concerning the association between AV and PROM. Han et al. found that pregnant women with AV were associated with a higher frequency for PROM (*p* < 0.05) [28]. Besides, a recent review article by Kaambo and Africa stated that even when asymptomatic, aerobic vaginitis may represent a risk factor for preterm delivery and PROM, ascending chorioamnionitis, and a neonatal mortality rate of 25%-90% because of congenital neonatal sepsis [29].

Our study has some limitations to consider. First, we screened pregnant women at late pregnancy (34-36 weeks). Earlier screening during pregnancy might detect more impacts of AV on perinatal outcomes. Second, the study was designed primarily to study only the association between AV and preterm labor and excluded other causes of vaginal infections. So, our study did not compare the impacts of AV to other forms of vaginal infections. Nonetheless, this study has the strength of being, to our knowledge, the first prospective powered study to evaluate the perinatal outcome of AV.

## 5. Conclusions

In conclusion, aerobic vaginitis is a common form of vaginal infection during pregnancy. AV is associated with a high incidence of preterm labor, PROM, and neonatal ICU admission. Moreover, the incidence of PTB and PROM might further increase with the severity of AV. Vaginal microbiota screening at 34-36 weeks might contribute to the improvement of pregnancy outcomes and reduction in preterm labor, PROM, and other adverse pregnancy outcomes. Additionally, clinicians should pay more consideration to vaginal microbiota assessment during pregnancy. Larger observational studies recruiting more patients of different ethnic populations are needed to support our findings and properly study all aspects of perinatal outcome.

## Figures and Tables

**Table 1:** Demographic and clinical characteristics.

Variable	AV (*n* = 100)	Control (*n* = 500)	*p* value
Age (years)	27.9 ± 0.57	27.2 ± 0.23	0.227∗
BMI (kg/m^2^)	28.1 ± 0.46	28 ± 0.2	0.964∗
Parity	1 (0-2)	1 (0-2)	0.125∗∗
Previous cesarean delivery	32 (32%)	154 (30.8%)	0.814^†^
Hypertensive disorders with pregnancy	4 (4%)	24 (4.8%)	1.0^‡^
Pregestational and gestational diabetes mellitus	7 (7%)	29 (5.8)	0.645^†^
Smoking habits	7 (7%)	33 (6.6%)	0.828^†^
Education level			<0.001^†^
High school or less	37 (37%)	66 (13.2%)	
College or above	63 (63%)	434 (86.8%)	
Woman's occupation			0.022^†^
Not working	34 (34%)	114 (22.8%)	
Working	66 (66%)	386 (77.2%)	

AV: aerobic vaginitis; BMI: body mass index. Data are presented as mean ± standard deviation, median (interquartile range), or number (%). ∗Student's *t*-test was used; ∗∗Mann-Whitney *U* test was used; ^†^Chi-squared test was used; ^‡^Fisher exact test was used. *p* value < 0.05 is significant.

**Table 2:** Associations of maternal outcomes among pregnant women with aerobic vaginitis and without aerobic vaginitis.

Variable	AV (*n* = 100)	Control (*n* = 500)	OR (CI)	*p* value	Adjusted∗ OR (CI)	*p* value
PTB	18 (18%)	34 (6.8%)	3.0 (1.62-5.58)	<0.001	3.06 (1.58-5.95)	0.001
PROM	24 (24%)	27 (5.4%)	5.53 (3.03-10.09)	<0.001	6.17 (3.24-11.7)	<0.001
pPROM	7 (7%)	21 (4.2%)	1.72 (0.71-4.16)	0.231	1.73 (0.68-4.4)	0.249
Chorioamnionitis	2 (2%)	3 (0.6%)	3.38 (0.56-20.5)	0.185	5.87 (0.9-38.24)	0.064
Cesarean delivery	32 (32%)	149 (29.8%)	1.11 (0.7-1.76)	0.662	1.11 (0.68-1.8)	0.679
Puerperal sepsis^†^	1 (1%)	0				

AV: aerobic vaginitis; OR: odds ratio; CI: confidence interval; PTB: preterm birth; PROM: prelabor rupture of membranes; pPROM: preterm prelabor mature rupture of membranes. Data are presented as number (%) and odds ratio with confidence interval. ∗The analysis was adjusted for maternal age, body mass index, parity, previous cesarean delivery, hypertensive disorders with pregnancy, pregestational and gestational diabetes mellitus, smoking habits, education level, and woman's occupation. ^†^As a result of the low frequency of this outcome, odds ratio was not reported. *p* value < 0.05 is significant.

**Table 3:** Associations of neonatal outcomes among pregnant women with aerobic vaginitis and without aerobic vaginitis.

Variable	AV (*n* = 100)	Control (*n* = 500)	OR (CI)	*p* value	Adjusted∗ OR (CI)	*p* value
Low birth weight	8 (8%)	19 (3.8%)	2.2 (094-5.18)	0.071	2.13 (0.85-5.4)	0.109
Neonatal jaundice	12 (12%)	38 (7.6%)	1.66 (0.83-3.3)	0.15	1.47 (0.7-3.09)	0.314
Neonatal sepsis	2 (2%)	2 (0.4%)	5.08 (0.71-36.51)	0.106	4.99 (0.6-41-53)	0.137
Neonatal asphyxia	5 (5%)	8 (1.6%)	3.24 (1.04-10.11)	0.043	2.9 (0.85-9.9)	0.089
NICU admission	15 (15%)	36 (7.2%)	2.28 (1.19-4.34)	0.013	2.19 (1.1-4.34)	0.025
Stillbirth	0	0				
Neonatal death	2 (2%)	1 (0.2%)	10.18 (0.91-113.4)	0.059	5.14 (0.3-86.69)	0.256

AV: aerobic vaginitis; OR: odds ratio; CI: confidence interval; NICU: neonatal intensive care unit. Data are presented as number (%) and odds ratio with confidence interval. ∗The analysis was adjusted for maternal age, body mass index, parity, previous cesarean delivery, hypertensive disorders with pregnancy, pregestational and gestational diabetes mellitus, smoking habits, education level, and woman's occupation. *p* value < 0.05 is significant.

**Table 4:** Comparison of maternal and neonatal outcomes according to the severity of aerobic vaginitis.

Variable	Severe AV (*n* = 24)	Mild/moderate AV (*n* = 76)	*p* value
PTB	10 (41.7%)	8 (10.5%)	0.0014
PROM	11 (45.8%)	13 (17.1%)	0.0094
pPROM	4 (16.7%)	3 (3.9%)	0.0549
Chorioamnionitis	1 (4.2%)	1 (1.3%)	0.4242
Cesarean delivery	9 (37.5%)	23 (30.3%)	0.4403
Puerperal sepsis	1 (4.2%)	0	0.240
Low birth weight	3 (12.5%)	5 (6.6%)	0.3688
Neonatal jaundice	3 (12.5%)	9 (11.8%)	0.2128
Neonatal sepsis	1 (4.2%)	1 (1.3%)	0.4242
Neonatal asphyxia	2 (8.3%)	3 (3.9%)	0.5910
NICU admission	6 (25%)	9 (11.8%)	0.2128
Neonatal death	1 (4.2%)	1 (1.3%)	0.4242

AV: aerobic vaginitis; PTB: preterm birth; PROM: prelabor rupture of membranes; pPROM: preterm prelabor rupture of membranes; NICU: neonatal intensive care unit. Data are presented as number (%). *p* value < 0.05 is significant. Severe aerobic vaginitis was diagnosed when a composite AV score > 6 was determined by saline wet mount microscopy [3].

## Data Availability

The data used to support the findings of this study are available from the corresponding author upon request.
